# Analysing the Relationship between University Students’ Ecological Footprint and Their Connection with Nature and Pro-Environmental Attitude

**DOI:** 10.3390/ijerph17238826

**Published:** 2020-11-27

**Authors:** Mónica Fernández, Gisela Cebrián, Elisa Regadera, M. Yolanda Fernández

**Affiliations:** 1 Department of Education, Universitat Internacional de Catalunya, Campus Sant Cugat, Josep Trueta, Sant Cugat del Vallès, 08195 Barcelona, Spain; 2 Department of Pedagogy, Universitat Rovira i Virgili, Campus Sescelades, 43007 Tarragona, Spain; gisela.cebrian@urv.cat; 3 School of Communication, Campus Barcelona, Inmaculada 22, Universitat Internacional de Catalunya, 08017 Barcelona, Spain; eregadera@uic.es; 4 Department of Social Sciences, Universidad Europea Miguel de Cervantes, Calle del Padre Julio Chevalier, 2, 47012 Valladolid, Spain; myfernandez@uemc.es

**Keywords:** ecological footprint, sustainable development goals, sustainability, environmental education, consumption

## Abstract

In the last decade, universities worldwide have adopted various measures intended to promote sustainability in higher education and include it in the curriculum. However, although this paradigm shift appears to be contributing to students’ acquisition of the knowledge, skills and values necessary to fight for a more sustainable world, serious global crises such as the present SARS-CoV-2 pandemic oblige us to rethink our behaviour and spur us to accelerate the move towards a deep-seated commitment to the environment and people. Therefore, the aims of this study were (a) to explore consumption habits in students at four Spanish universities by analysing their individual ecological footprint (EF); (b) to develop indices of connection with nature and a pro-environmental attitude and to determine relationships between these indices and students’ consumption. Among other factors, our results showed that private university students have a higher EF than public university students; that food consumption has the greatest impact on individuals’ EF; and that those who consume more sustainably do not show a more pro-environmental attitude or feel a greater connection with nature. Therefore, we conclude that there was no apparent direct relationship between study participants’ convictions and their daily behaviour. There is a pressing need in education to demonstrate the connection between our actions and their environmental impact.

## 1. Introduction

The UN’s declaration of its Sustainable Development Goals (SDG) in 2015 and the Conference on Climate Change in the same year constituted two important milestones that have since determined the environmental and educational policies and actions of governments, companies and institutions. The United Nations document, the “2030 Agenda” [1], contains a series of integrated and indivisible objectives and goals that combine three dimensions—economic, social and environmental—considered essential for sustainable development. Previously, authors such as Tilbury [2] had also indicated that contemporary cultural change is leading towards the adoption of an integrated approach to sustainable development through public engagement and the implementation of measurable frameworks in governance, research, teaching, management and operations.

In the field of education, 2015 marked the end of the UN Decade of Education for Sustainable Development (DESD, 2005–2014), which was aimed at inserting the principles, values and practices of sustainable development into all aspects of education and learning. In 2016, the UN Economic Commission for Europe [3] reviewed implementation of sustainability goals and produced an evaluation report of the work carried out in the previous decade, emphasising the crucial importance of education in contributing to development of the knowledge, skills and values necessary to create a more sustainable world [4].

However, higher education continues to pose the greatest challenge in the field of education. In the 1990s, numerous measures were declared to promote sustainability in universities [5,6,7] by incorporating the concept into all university courses [8]. However, despite these efforts, environmental and economic questions still tend to be considered separately rather than together [9,10,11] contrary to UN guidance [4]. Numerous universities around the world have committed to sustainability through the adoption of international declarations, the creation of sustainability strategic plans and the inclusion of one or several Sustainable Development Goals (SDGs) within specific undergraduate and master’s degree programmes [12]. However, this does not necessarily translate into real practice and have an influence on students’ engagement, learning and behavioural change towards sustainability [13].

Notable among the initiatives implemented to achieve these educational and awareness raising objectives was the guide produced in 2017 by the Australia, New Zealand and Pacific Network of the Sustainable Development Solutions Network (SDSN), which explains how universities can engage with the SDGs. The guide notes the lack of previous guidance material and the difficulties universities encounter in leveraging existing resources, but also stresses the need to draw on the experiences of other universities that are already working in this direction. University teachers have also needed to engage with this paradigm shift, which not only implies developing sustainability competencies, but also innovating through appropriate instruction for an education in sustainable development. This task involves countless challenges, including the need to promote relational thinking, improve the contextualisation of teaching and carry out practical work consistent with theoretical proposals [14].

It is widely accepted that the present environmental crisis has mainly been caused by the West’s model of consumer behaviour [15,16]. Is this environmental crisis related to the emergence of the new type of coronavirus? Numerous authors have related the unprecedented health and socio-economic crisis caused by the SARS-CoV-2 pandemic to factors such as industrial livestock production and ecosystem change caused by monoculture, intensive deforestation and the massive generation and interaction of pollutants and highly complex, toxic chemicals dispersed in the environment [17,18,19]. To a greater or lesser extent, scientists link the current pandemic to the destruction of ecosystems caused by a capitalist food system [20]. If production and consumption models are not changed soon, people will be exposed to new pandemics and will be vulnerable to new health, social and economic crises [21].

Halting planetary devastation is crucial, and this will involve changing the lifestyles of the younger generations, who are the main consumers [22]. Since education is one of the fundamental tools for shifting society towards a more sustainable model, Albareda [22] has suggested that universities have a particular responsibility to actively contribute to an education for sustainable consumption among young people. This should involve all stakeholders’ engagement to create a common shared vision on how to implement sustainability in university teaching, as well as promote research and dissemination of sustainability knowledge [12].

All this confirms that we must continue moving towards a paradigm shift in education, seeking the appropriate methodologies and tools while also bearing in mind that it is not simply a question of transferring responsibility to teachers and institutions. We must transform the consumption habits and personal attitudes of students by re-orientating the curriculum and better addressing the needs of current and future generations [4]. In this context, some teachers and researchers have used the ecological footprint tool to raise university students’ awareness [14]; the aim is to change students’ consumption habits through increased knowledge, analysis and reflection on the planetary impact of their consumption.

Several NGOs provide online tools to calculate one’s individual EF, but Fernández et al. [14] selected the one designed by the organisation Redefining Progress because of its advantages over other tools: it offers the option of selecting a country; gives average figures per inhabitant; calculates global EF and EF by category (carbon, food, goods and services, housing); and provides information and recommendations on the most sustainable alternatives in each case.

An education for sustainability that examines individuals’ EF seems to promote consumer change, and not only in terms of reducing the carbon footprint. One study has shown that, when consumers are already engaging in sustainable behaviour, this can be extended to other, more difficult actions, producing a positive spill-over effect as a means to increase sustainable choices [23].

Despite its potential, the online EF tool also has limitations: it provides little information about its methods and estimates [24,25], but nevertheless appears suitable for use by university students because it presents their individual behaviour in the format of a very clear, informative image.

Thus, the objectives of this study were twofold:

(a) To analyse the ecological footprint of university students taking a degree in education at four Spanish universities, two public (the University of Seville and the University of Cádiz) and two private (the Camilo José Cela University and the International University of Catalonia).
(b) To create indices measuring students’ pro-environmental attitude and connection with nature in order to investigate the possible relationship between consumption and environmental attitudes and feelings.

## 2. Materials and Methods 

### 2.1. Sample

This study was conducted in the academic year 2018–2019 with students taking a degree in primary or pre-school education at four Spanish universities. Table 1 shows the sample obtained in each case.

### 2.2. Data Collection and Analysis Instruments

Previous works such as that of Chuvieco et al. [26] included a questionnaire of 25 sustainable habits of students, following a Likert scale from 1 (never) to 5 (always). Likewise, in a complementary way, the students were asked about their environmental concern on a scale of low, medium and high. The work presented here includes an approach to the research objectives similar to that followed by these authors, but consumption habits were converted into personal Ecological Footprint by means of an online calculator, obtaining a value for each student of their global EF in global hectares (gh), as well as the EF broken down into categories: Carbon Footprint (CF), Food Footprint (FF), Goods and Services Footprint (GSF) and Housing Footprint (HF). The online calculator selected from those provided by many NGOs and those available on the Internet was the one offered by the organisation Redefining Progress (RP) of the Center for Sustainable Economy [27], because of the advantages explained in a previous work [14]. Table 2 shows the definition of each EF category (CF, FF, GSF and HF) as well as the indicators used in the online RP programme for calculating them.

To determine the students’ connection with nature and pro-environmental attitude, we administered a questionnaire of nine questions with four response categories (never, almost never, sometimes and always) based on the Nature Relatedness Scale (NR-6) developed by Nisbet and Zelenski [28]; to the six questions included in this questionnaire on “thoughts, wishes or feelings”, three more about “actions” were added (Table 3). Thereby this questionnaire allows us to explore what Chubieco et al. [26] call environmental concern in more depth.

Data were analysed using the IBM software package SPSS v. 26 IBM Corp. (Armonk, NY, USA) for Windows. The treatment of these data included an exploratory factor analysis to identify complex interrelationships between items that are part of unified concepts, finding two factors, but revealing that two of the variables had little correlation with the rest (which were eliminated). Subsequently, a confirmatory factor analysis was carried out and a bifactorial structure with high scores and high internal consistency was reaffirmed (Cronbach’s alpha).

With the results obtained, two indices using an ordinal scale were constructed to analyse, with non-parametric contrasts, if there were significant differences between the universities.

## 3. Results

### 3.1. Students’ Individual EF

Table 4 shows the results obtained for EF expressed in global hectares (gha) and disaggregated by category: Food Footprint (FF), Goods and Services Footprint (GSF), Carbon Footprint (CF) and Housing Footprint (HF). The table also shows the average national values provided by the tool.

These figures indicate that, in agreement with the national average, the largest contribution in all cases to global EF came from the food footprint (FF), while the smallest contribution came from the housing footprint (HF). However, in all cases the global EF was lower than the national average.

**Figure 1:**
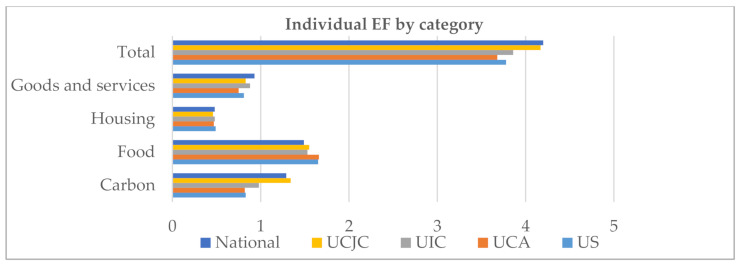
Results (in gh) for individual environmental footprint (EF) disaggregated by category and compared to the national average (the data of the Ecological Footprint of each student is provided as supplementary material in Table S1).

These results are consistent with the fact that students fall below the national average in almost all categories, with the following exceptions: the FF was higher among university students—from any of the universities analysed—than among the general population, while the CF was higher than the national average solely in the case of UCJC students, with a notable difference to the rest of the universities (Figure 1).

As can be seen, students’ EF was higher in private than in public universities analysed; the UCJC headed the list, followed in descending order by the UIC, the US and the UCA.

### 3.2. Connection with Nature and Pro-Environmental Attitude

The results obtained from the questionnaire (Table 3) are given in detail in Table 5.

Of all the results obtained, the following aspects merit particular attention:

- None of the students from the US or the UCJC stated that they would always choose a wild, remote place for their holidays (question 1).
- None of the students from the US stated that they always thought about how their actions affected the environment (question 2).
- None of the students from any of the universities said that they always read news related to the environment (question 8).
- None of the students from the UIC, US or UCA stated that they always participated in pro-environmental actions or conservation activities (questions 7 and 9).

### 3.3. Development of Indices to Measure Pro-Environmental Attitude and Connection with Nature

We performed a statistical analysis of the data obtained in order to create indicators that would measure actions representing concern, interest and active involvement in environmental protection and conservation (termed a pro-environmental attitude) and the feeling of belonging to and connection with the natural environment (here termed connection with nature).

#### 3.3.1. Factor Analysis

First, we conducted a factor analysis of the principal components in order to construct indices that would allow comparisons between the four universities.

The nine variables were subjected to an exploratory factor analysis, revealing that the variable “I read about environmental issues” obtained a very low score for the factors obtained. It was therefore eliminated. One explanation for this may be the way in which the question was phrased: “I read” seems to suggest books or written texts, whereas “I consult” might have been interpreted to refer to any source, including digital. Meanwhile, the variable “I think about how my actions affect the environment” obtained the second lowest score, and furthermore presented a negative or very low correlation with the other variables. Consequently, it was also eliminated from the study.

A confirmatory principal component factor analysis was performed with the remaining seven variables, obtaining a clear factor structure in which all items presented a value of ≥0.5 [29] (Table 6), i.e., all variables were very well represented in the factor space.

To simplify the matrix and facilitate interpretation of the results, we then performed an orthogonal rotation (Varimax). The Kaiser-Meyer-Olkin (KMO) test yielded a value of 0.807, which can be considered excellent, and Bartlett’s X2 test was statistically significant (*p* = 0.000), explaining 63% of the variance, indicating that the results of the analysis could be considered satisfactory [30] (Table 7).

In the light of the results, we then identified the factors to take into account, in order to determine a connection with nature and a pro-environmental attitude.

Of the seven variables analysed, four (I feel connected to nature, I feel happy in nature, my relationship with nature is important to me and I feel concerned about living beings and the Earth) were grouped into one factor (with a Cronbach’s alpha of 0.792), while the remaining three (I would choose a wild, remote place for my holidays, I take part in pro-environmental actions, I take part in conservation activities) were grouped into another with a Cronbach’s alpha of 0.634 (Table 8).

**Figure 2:**
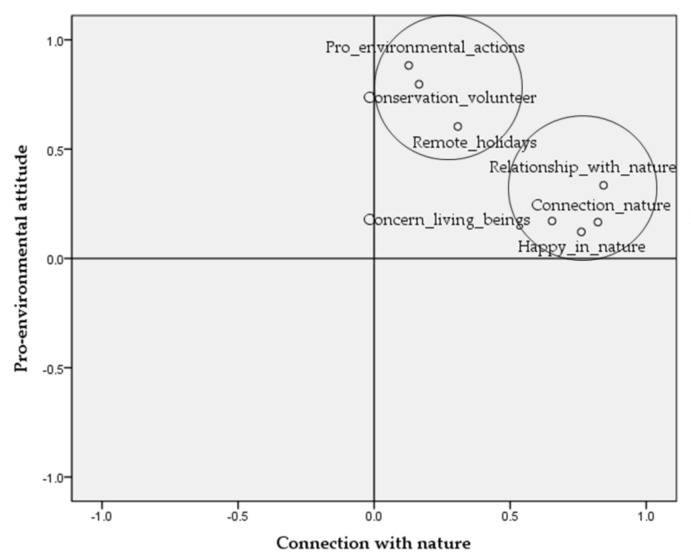
Rotated components.

Figure 2 of rotated components shows how the variables scored for each of the factors.

#### 3.3.2. Calculation of Indices and Comparison between Universities

These results were used to create two indices to measure: (1) pro-environmental attitude and (2) connection with nature. To this end, the seven variables were recoded so that the responses “never” and “almost never” were assigned the value of zero and the responses “sometimes” or “always” were assigned the value of 1. This enabled us to create both indices by adding together the values of the recoded variables that obtained a score for each of the factors. Values for index 1 ranged from zero to three, while values for index 2 ranged from zero to four.

##### Index 1 = Pro-Environmental Attitude

The mean for this index was 1.13 (SD = 0.89), and the Kruskal Wallis H test revealed significant differences between universities (X2 (4) = 15.756, *p* = 0.001). It should be noted that 12 students from the UIC did not respond to the first question in the questionnaire (I would choose a wild, remote place for my holidays), and it was therefore not possible to calculate the pro-environmental attitude index for these students (Table 9).

The university with the highest pro-environmental attitude index was the UCJC, followed by the UCA, UIC and US. A paired Mann-Whitney U test revealed significant differences between the US and the UCJC (X2 (2) = 352.500, *p* = 0.000), the UIC and the UCJC (X2 (2) = 229.500, *p* = 0.049) and the US and the UCA (X2 (2) = 1380, *p* = 0.011) (Table 10).

##### Index 2 = Connection with Nature

The mean for the index “connection with nature” was 3.0757 (SD = 1.19), and the Kruskal Wallis H test revealed significant differences between universities (X2 (4) = 42.893, *p* = 0.000). The university that obtained the highest connection with nature index was the UIC, closely followed by the UCJC and UCA, and less closely by the US (Table 11).

A paired Mann-Whitney U test revealed significant differences between the US and all the other universities: UIC (X2 (2) = 542.500, *p* = 0.000), UCA (X2 (2) = 882.500, *p* = 0.000) and UCJC (X2 (2) = 311.000, *p* = 0.000) (Table 8).

### 3.4. Relationship between Individual Consumption (EF) and the Indices Developed

The findings reported in the above sections indicate that:

1. The UCJC presented the highest pro-environmental attitude index and showed statistically significant differences with the US, which obtained the lowest index; however, UCJC students also obtained the highest global EF, which was, furthermore, the only case in which student EF exceeded the national average.
2. The UIC presented the highest connection with nature index while the US once again obtained the lowest index, with statistically significant differences to all the other universities. However, the global EF of students at private universities was higher than that of those at public ones, with the UIC in second place.
3. Students at this latter university showed the lowest individual consumption (EF) and occupied intermediate positions in the indices calculated.

## 4. Discussion

The literature on sustainability in higher education evidences the efforts made in this field in recent decades by universities and researchers to conceptualise and measure the ecological footprint of students and university teachers alike and to improve environmental management of university campuses [14,31,32,33,34].

As the results of the present study show, all the students, regardless of the university they were attending, were below the national EF in all categories except food footprint. This finding may indicate the impact of good practice in ESD at the universities, the inclusion of sustainability in specific courses and in different subject areas such as engineering, life sciences, business studies or education, and the effect of the eleven declarations, charters and partnerships for sustainability in higher education [35,36,37]. Chuvieco et al. [26] analysed the environmental habits of university students in Spain, Brazil and the United Arab Emirates. The results showed that students’ sustainability habits were influenced by the subject area of study and self-perceived environmental commitment, while no relevance was found in relation to the year of study.

According to a recently conducted study in Portugal [12] universities have in general showed their commitment through the integration of the SDGs in different undergraduate and postgraduate programmes. However, to date, university stakeholders perceive sustainability implementation differently, which can represent a challenge in achieving the holistic transformation advocated by ESD scholars [2,38].

The results regarding the contribution of the different EF categories are similar to those obtained by Collins et al. [39] in other public European universities (in the UK and Italy). These authors found that the footprint category with the highest level of resource consumption was food, followed by goods and services and then mobility (which would be equivalent to the carbon footprint according to our calculator). In relation to the universities analysed here, our results indicate that students at private universities had a higher EF than those at public ones; the UCJC (Madrid) headed the list, followed by the UIC (Barcelona) and then the US and the UCA (both in Andalusia). Paradoxically, however, the UCJC was also the university with the highest pro-environmental attitude index, followed by the UCA, UIC and US. In other words, those students who presented less sustainable consumption according to their calculated EF were the same ones who reported having a more pro-environmental attitude. This indicates, according to existing studies [26], that universities, apart from promoting sustainability knowledge, should impact on changing behaviours and mindsets amongst students.

In this respect, income level has been positively correlated with pro-environmental behaviour, while previous studies have found that socio-demographic and socio-economic variables may also be associated with a higher EF [40,41]. Furthermore, residents of urban areas have a higher EF [42]. Although no current information is available on EF by region in Spain, the most recent data, obtained for the period 1995–2005 [43], indicate that of the 17 autonomous regions, those with the highest EF in Spain are Madrid (UCJC), Catalonia (UIC) and Andalusia (UCA and US), all regions in which a high percentage of the population resides in urban centres. Coinciding with these data, several studies [44,45] found that in urban households in China the Blue Water Footprint (BWF) and the Grey Water Footprint (GWF) are higher than in rural households. Thus, blue water consumption per capita in urban households is more than 40% higher than in rural households. Although BWF and GWF have a positive correlation with income, their marginal growth diminishes because of certain influencing factors such as consumption patterns. For instance, rich households are inclined to spend most of their money on education and recreation rather than on food (food consumption dominated household water footprint).

It is extremely worrying that the very students who felt most connection with nature (UIC) or who showed the strongest pro-conservation attitudes or reported the most happiness in nature (UCJC) were precisely the ones who had the worst impact on the environment as a result of their consumption habits. These contradictions in young university students have also been reported in other studies, in which they were found to simultaneously display pro-environmental attitudes and anti-environmental behaviour [46]. Other studies [13] also show that campus sustainability and environmental information significantly determined students’ involvement in sustainability. According to Salazar and Portillo [47], the relationship and interaction between ecological knowledge and pro-environmental attitudes must be strengthened through education, especially with regard to waste management, biodiversity, pollution, recycling and energy, since this information determines attitudes. On the other hand, the students’ expressed connection with nature could be interpreted as reflecting a more anthropocentric and functional vision rather than an eco-centric one in which nature is considered beautiful but fragile [48], which would explain the pressure exerted on nature through heedless consumption of resources.

As a limitation of this study, we should mention the need to include other data collection methods of a more qualitative nature, such as interviews, focus groups or observation, since other studies of sustainability in higher education have indicated that surveys and questionnaires reflect self-perceived levels rather than actual behaviour, and each student may interpret the indicators in different terms [49]. Questionnaires may also present greater self-perception, which can differ from actual behaviour [50,51].

ESD involves promoting cognitive, socio-emotional and behavioural learning objectives [52]. A variety of tools must be used to evaluate such learning in order to enable students to reflect on the value of sustainability both personally and professionally [53]. Therefore, a combination of quantitative and qualitative assessment tools is required, including reflective journals, interviews, focus groups, observation, and case- or problem-solving, in order to triangulate perception, capacity and behaviour when determining an individual’s ecological footprint and environmental behaviour. Future research should include longitudinal studies, mixed methods, and pre-post test designs with control groups, in order to compare quantitative results and qualitative assessment of behaviour.

It should be noted that research on individuals’ ecological footprint and the determinants of the environmental impact of some behaviours has only recently emerged. Most studies have focused on exploring differences between countries [54,55] and few studies have explored individuals’ ecological footprint [41]. Nevertheless, recent years have witnessed an increase in studies measuring university students’ ecological footprint [56,57], which indicates the contribution of these calculators in determining the environmental awareness underpinning behaviours related to consumption habits and sustainable practices in universities [32].

## 5. Conclusions

In relation to the first study objective, our university students showed a lower individual EF than the national average, and in common with the general population their worst environmental impact was related to their food consumption. These findings are in agreement with the results of previous studies conducted in Spain and other European countries, indicating that further education is required at individual level to change food consumption habits. Our findings also seem to support the idea that a high socio-economic level and residence in urban settings are related to greater consumption, since a higher EF was obtained at the two private universities analysed, located in Madrid and Barcelona. Students need to apply their ecological knowledge to their consumption decisions, especially at this critical time when a relationship has been posited between food consumption, ecosystem destruction and the COVID-19 pandemic.

With regard to the other objective (the development of a pro-environmental attitude and connection with nature indices and their relationship with personal consumption), we found no apparent relationship between more sustainable habits (smaller EF) and a greater connection with nature or a pro-environmental attitude. Students who perceived themselves to have a greater connection with nature and expressed pro-protection attitudes were in fact those who obtained a higher EF. Once again, this evidences the urgent need for educational interventions to instil an awareness in students that our individual actions have global repercussions, and our consumption is directly linked to the use of resources and the destruction of nature and its ecosystems. Only thus can we prevent future environmental and/or health crises such as the present one, and this will demand greater commitment not only at institutional or administrative level but also, as shown here, at individual level.

## Figures and Tables

**Table 1:** Participants from each university.

**University**		**Participants** **EF/Questionnaire**
Private	International University of Catalonia (UIC Barcelona)	53/41
	Camilo José Cela University (UCJC)	19/23
Public	University of Cádiz (UCA)	52/60
	University of Seville (US)	42/61
Total		166/185

**Table 2:** Definitions and indicators used in the Redefining Progress programme for calculating Footprints (table adapted from Fernández et al. [14]).

Food Footprint (FF)	Housing Footprint (HF)
Definitions	
“The amount of cropland, pastureland, and marine fisheries supporting annual food consumption plus the land and ocean area required to absorb carbon emission associated with food production, processing and transportation”	“The area needed to replace the resources used for housing construction and maintenance”
Indicators	
Type of diet Preferred place for buying Frequency of purchasing organic or sustainably produced food Volume of food consumed and temporal distribution of meals throughout the day To grow vegetables or herbs at home or on allotments	Type of accommodation Building material used for housing Source of furnishings Type of cleaning products used Water-saving devices and habits employed
**Carbon Footprint (CF)**	**Goods and Services Footprint (GSF)**
Definitions	
“The amount of land and ocean area required to absorb carbon emission associated with an individual’s home energy use and transportation”	“The area of biologically productive land and water required to assimilate the wastes generated as a result of spending habits, waste disposal, recycling behavior and clothing and paper product choices”
Indicators	
Climate of the area in which the population sample lives Size of accommodation Location of accommodation Domestic energy sources Percentage of electricity generated from renewable energy sources Annual distance in kilometers travelled in all means of transport Domestic energy-saving devices and habits	Amount of expenditure and savings Frequency of replacement of objects Volume of waste generated Percentage and type of recycled items: paper, aluminium, glass, plastic, electronic goods… Frequency of purchasing stationery and/or articles of clothing which are labelled “organic” or are made sustainably

**Table 3:** Ad hoc questionnaire based on that developed by Nisbet and Zelenski (2013).

Questions	N	AN	S	A
1. Would you choose a wild, remote place for your holidays?				
2. Do you think about how your actions affect the environment?				
3. Do you feel a connection with nature and the environment?				
4. Do you feel happy when out in nature?				
5. Is your relationship with nature important to you?				
6. Are you concerned about living beings and the Earth?				
7. Do you take part in pro-environmental actions?				
8. Do you read about environmental issues in the media?				
9. Do you take part in conservation actions?				

Note: N: never; AN: almost never; S: sometimes; A: always.

**Table 4:** Global EF and disaggregated by category, for education (primary or pre-school) degree students at four Spanish Universities: UIC (Barcelona), US (Seville), UCA (Cádiz) and UCJC (Madrid). The data of the global EF an disaggregated bay category of each student is provided as supplementary material (Table S1).

		CF	FF	HF	GSF	Global EF
UIC (N = 53)	A (gha)	0.97	1.52	0.47	0.87	3.77
	SD	0.64	0.32	0.12	0.23	0.83
US (N = 42)	A (gha)	0.82	1.64	0.48	0.80	3.72
	SD	0.27	0.32	0.12	0.28	0.68
UCA (N = 52)	A (gha)	0.83	1.65	0.46	0.73	3.67
	SD	0.37	0.28	0.12	0.26	0.52
UCJC (N = 19)	A (gha)	1.33	1.54	0.45	0.83	4.17
	SD	1.23	0.29	0.14	0.24	1.27

Note: A = average, SD = standard deviation, gha: global hectares.

**Table 5:** Results obtained from the questionnaire (Table 3) on students’ connection with nature and pro-environmental attitude. Data expressed as a percentage of responses.

Question	UCJC (*n* = 23)				UIC (*n* = 41)				UCA (*n* = 60)				US (*n* = 61)			
	A	S	AN	N	A	S	AN	N	A	S	AN	N	A	S	AN	N
1.	0	66	20	8	20	55	13	10	10	58	23	8	0	62	24	9
2.	20	54	16	8	9	78	12	0	15	78	0	0	0	67	27	0
3.	33	37	20	8	17	56	19	7	21	43	30	0	11	27	44	16
4.	66	29	0	0	75	24	0	0	75	25	0	0	19	45	29	0
5.	54	25	16	0	39	48	0	0	35	46	16	0	13	24	45	16
6.	62	33	0	0	56	41	0	0	68	31	0	0	18	55	21	0
7.	16	33	25	25	0	19	39	41	0	35	36	23	0	13	41	44
8.	0	33	37	25	0	22	34	39	0	31	33	33	0	13	41	42
9.	8	33	20	37	0	9	24	63	0	13	23	60	0	0	18	82

Note: A = always; S = sometimes; AN = almost never.

**Table 6:** Multiple linear correlation coefficient of each variable with the factors.

	Initial	Extraction
Connection to nature	1.000	0.606
Happy in_nature	1.000	0.668
Relationship_with_nature	1.000	0.765
Concern_for living_beings	1.000	0.562
Pro-environmental_actions	1.000	0.779
Conservation_volunteer	1.000	0.736
Remote_holidays	1.000	0.499

Note: Method of extraction: principal component analysis.

**Table 7:** KMO and Bartlett’s test.

Kaiser-Meyer-Olkin Measure of Sampling Adequacy		0.807
Bartlett’s test of sphericity	Approximate chi-square	373.308
	gl	21
	Sig.	0.000

**Table 8:** Rotated component matrix.

Variables	Component	
	1	2
Connection_with nature	0.741	0.232
Happy_in_nature	0.818	0.117
Relationship_with_nature	0.794	0.367
Concern_for living_beings	0.754	0.120
Pro-environmental_actions	0.124	0.829
Conservation_volunteer	0.162	0.800
Remote_holidays	0.240	0.558

Note: Extraction method: principal component analysis. Rotation method: Varimax with Kaiser normalisation. The rotation converged in three iterations.

**Table 9:** Descriptive indices for pro-environmental attitude.

University	N	Index Mean	Standard Deviation	Minimum	Maximum
US	61	0.8033	5.7212	0	2
UCA	60	1.2500	0.9677	0	3
UIC	29	1.1379	0.8752	0	3
UCJC	23	1.6957	1.0632	0	3

**Table 10:** Mann-Whitney U Test.

Paired Groupings	Mann-Whitney U Test	Asymptotic sig. (Bilateral)
US-UCA	1380	**0.011** *
US-UIC	703.500	0.078
US-UCJC	352.500	**0.000** ***
UCA-UIC	824.000	0.671
UCA-UCJC	519.500	0.071
UIC-UCJC	229.500	**0.046** *

Note: *: *p* < 0.05; ***: *p* < 0.001.

**Table 11:** Descriptive indices for connection with nature.

UNIVERSITY	N	Mean	Standard Deviation	Minimum	Maximum
US	61	2.1639	1.45121	0	4
UCA	60	3.4667	0.65008	2	4
UIC	41	3.5854	0.66991	2	4
UCJC	23	3.5652	0.78775	2	4

## Supplementary Materials

The following are available online at https://www.mdpi.com/1660-4601/17/23/8826/s1, Table S1: Global EF and disaggregated bay category of each student. Because the website www.myfootprint.org is not available at this time due to some problems (see: https://www.sustainable-economy.org/myfootprint.html), the excel with the data of the Ecological Footprint of the students is provided as supplementary material.

## References

[1] United Nations (UN) (2015). Transforming our World: The 2030 Agenda for Sustainable Development. Resolution Adopted by the General Assembly on 25 September 2015 (A/70/L.1). http://sustainabledevelopment.un.org/post2015/transformingourworld.

[2] Tilbury D. Peoples’ Sustainability Treaty on Higher Education Draft for Rio+20. Proceedings of the United Nations Conference on Sustainable Development, Rio+20.

[3] UNECE (2012). Learning for the Future: Competences in Education for Sustainable Development.

[4] Albareda S., Fernández M., Mallarach J.M., Vidal S. (2017). Barreras para la sostenibilidad integral en la universidad. Rev. Iberoam. Educ..

[5] Leal W. (2010). Teaching sustainable development at university level: Current trends and future needs. J. Balt. Sci. Educ..

[6] Lozano R., Lukman R., Lozano F.J., Huisingh D., Lambrechts W. (2013). Declarations for sustainability in higher education: Becoming better leaders, through addressing the university system. J. Clean. Prod..

[7] Michelsen G., Barth M., Michelsen G., Rieckmann M., Thomas I. (2016). Policy, Politics and Polity in Higher Education for Sustainable Development. Routledge Handbook of Higher Education for Sustainable Development.

[8] Wals A.E. (2012). Shaping the Education of Tomorrow: 2012 Report on the UN Decade of Education for Sustainable Development.

[9] Bond J., Morrison-Saunders A. (2011). Re-evaluating sustainability assessment: Aligning the vision and the practice. Environ. Impact Assess. Rev..

[10] Fien J., Tilbury D., Tilbury D., Stevenson R.B., Fien J., Schreuder D. (2002). The Global Challenge of Sustainability. Education and Sustainability: Responding to the Global Challenge.

[11] Pérez D.G., Vilches A., Oliva J.M. (2005). Década de la educación para el desarrollo sostenible. Algunas ideas para elaborar una estrategia global. Eureka.

[12] Aleixo A., Azeiteiro U.M., Leal Filho W. (2020). Are the sustainable development goals being implemented in the Portuguese High. Education formative offer?. Int. J. Sustain. High. Educ..

[13] Dagiliuté R., Liobikienė G., Minelgaitė A. (2018). Sustainability at Universities: Student´s Perceptions from Green and Non-Green Universities. J. Clean. Prod..

[14] Fernández M., Alférez A., Vidal S., Fernández M., Albareda S. (2016). Methodological approaches to change consumption habits of future teachers in Barcelona, Spain: Reducing their personal Ecological Footprint. J. Clean. Prod..

[15] Sanyé-Mengual E., Secchi M., Corrado S., Beylot A., Sala S. (2019). Assessing the decoupling of economic growth from environmental impacts in the European Union: A consumption-based approach. J. Clean. Prod..

[16] IPCC (2018). Global Warming of 1.5 °C. IPCC Special Report. https://www.ipcc.ch/site/assets/uploads/sites/2/2019/06/SR15_Full_Report_High_Res.pdf.

[17] Luna-Nemecio J.M. (2020). Determinaciones socioambientales del COVID-19 y vulnerabilidad económica, espacial y sanitario-institucional. Rev. Cien. Soc..

[18] Everard M., Johnston P., Santillo D., Staddon C. (2020). The role of ecosystems in mitigation and management of Covid-19 and other zoonoses. Environ. Sci. Policy.

[19] Manoj M.G., Kumar M.K., Valsaraj K.T., Sivan C., Vijayan S.K. (2020). Potential link between compromised air quality and transmission of the novel corona virus (SARS-CoV-2) in affected areas. Environ. Res..

[20] Espejo W., Celis J.E., Chian G., Bahamonde P. (2020). Environment and COVID-19: Pollutants, impacts, dissemination, management and recommendations for facing future epidemic threats. Sci. Total Environ..

[21] Benach J. (2020). We Must Take Advantage of This Pandemic to Make a Radical Social Change: The Coronavirus as a Global Health, Inequality, and Eco-Social Problem. Int. J. Health Serv..

[22] Albareda S., Leal Filho W., Azul A., Brandli L., Özuyar P., Wall T. (2019). Empowering and Mobilizing Youth for SDG 12. Responsible Consumption and Production. Encyclopedia of the UN Sustainable Development Goals.

[23] Penz E., Hartl B., Hofmann E. (2019). Explaining consumer choice of low carbon footprint goods using the behavioral spillover effect in German-speaking countries. J. Clean. Prod..

[24] Franz J., Papyrakis E. (2011). Online calculators of ecological footprint: Do they promote or dissuade sustainable behaviour?. Sustain. Dev..

[25] Cucek L., Klemes J., Kravanja Z. (2012). A Review of Footprint analysis tools for monitoring impacts on sustainability. J. Clean. Prod..

[26] Chuvieco E., Mario B., Da Silva E.V., Hussein K., Alkaabi K. (2018). Factors affecting environmental sustainability habits of university students: Intercomparison analysis in three countries (Spain, Brqzil and UAE). J. Clean. Prod..

[27] Redefining Progress. Center for Sustainable Economy. My Ecological Footprint Quiz, n.d.. www.myfootprint.org.

[28] Nisbet E., Zelenski J. (2013). The NR-6: A new brief measure of nature relatedness. Front. Phycol..

[29] Nunnally J.C., Bernstein I.H. (1994). Psychometric Theory.

[30] D´Ancona M.A. (2002). Análisis Multivariable. Teoría y Práctica en la Investigación Social.

[31] Alves-Pinto M.J., Giannetti B.F., Leal Filho W., Bardi U. (2019). Sustainable Universities: A Comparison of the Ecological Footprint, Happiness and Academic Performance among Students of Different Courses. Sustainability on University Campuses: Learning, Skills Building and Best Practices.

[32] Collins A., Galli A., Patrizi N., Pulselli F. (2018). Learning and teaching sustainability: The contribution of Ecological Footprint calculators. J. Clean. Prod..

[33] Lambert M., Cushing K.K. (2017). How low can you go? Understanding ecological footprint reduction in university students, faculty and staff. Int. J. Sustain. High. Educ..

[34] Südaş H.D., Ozeltürkay E.Y. (2015). Analyzing the Thoughts of Ecological Footprints of University Students: A Preliminary Research on Turkish Students. Procedia Soc. Behav. Sci..

[35] Barth M., Rieckmann M., Barth M., Michelsen G., Rieckmann M., Thomas I. (2016). State of the art in research on higher education for sustainable development. Routledge Handbook of Higher Education for Sustainable Development.

[36] Cebrián G., Grace M., Humphris D. (2015). Academic staff engagement in education for sustainable development. J. Clean. Prod..

[37] Wright T.S.A. (2010). University presidents’ conceptualizations of sustainability in higher education. Int. J. Sustain. High. Educ..

[38] Aleixo A., Azeiteiro U.M., Leal Filho W., Leal Filho W., Azeiteiro U.M., Alves F., Molthan-Hill P. (2017). UN decade of education for sustainable development: Perceptions of higher education Institution’s stakeholders. Handbook of Theory and Practice of Sustainable AQ: 5 Development in Higher Education.

[39] Collins A., Galli A., Hipwood T., Murthy A. (2020). Living within a One Planet reality: The contribution of personal Footprint calculators. Environ. Res. Lett..

[40] Druckman A., Jackson T. (2009). The carbon footprint of UK households 1990–2004: A socio-economically disaggregated, quasi-multi-regional input-output model. Ecol. Econ..

[41] Kennedy E.H., Krahn H., Krogman N.T. (2015). Are we counting what counts? A closer look at environmental concern, pro-environmental behavior, and carbon footprint. Local Environ..

[42] Sovacool B., Brown M. (2010). Twelve metropolitan carbon footprints: A preliminary comparative global assessment. Energy Policy.

[43] Spanish Ministry of the Environment, Rural and Marine Affairs (2008). Análisis de la Huella Ecológica de España. Sostenibilidad y territorio. Gobierno de España. http://movil.asturias.es/medioambiente/articulos/ficheros/Huella%20ecologica%20de%20Espana.pdf.

[44] Liao X., Chai L., Liang Y. (2021). Income impacts on household consumption’s grey water footprint in China. Sci. Total Environ..

[45] Chai L., Han Z., Liang Y., Su Y., Huang G. (2020). Understanding the blue water footprint of households in China from a perspective of consumption expenditure. J. Clean. Prod..

[46] Sandoval-Escobar M., Páramo P., Orejuela J., Gonzáles-Gallo I., Cortés O.F., Herrera-Mendoza K., Garzón C., Erazo C. (2019). Paradoxes of the environmental behavior of university students in different academic disciplines. Interdisciplinaria.

[47] Salazar J.A., Portillo J. (2019). Relationship between pro-environmental attitudes and ecological knowledge in adolescents in relation to the rural or urban environment they inhabit. Kavilando.

[48] Pasca L. (2019). Nature, Connectedness and Well-Being. International Doctoral Thesis.

[49] Shephard K., Harraway J., Lovelock B., Mirosa M., Skeaff S., Slooten L., Strack M., Furnari M., Jowett T., Deaker L. (2015). Seeking learning outcomes appropriate for ‘education for sustainable development’ and for higher education. Assess. Eval. High. Educ..

[50] Cebrián G., Pascual D., Moraleda A. (2019). Sustainability competencies amongst Spanish pre-service secondary school teachers. Int. J. Sustain. High. Educ..

[51] Sandri O., Holdsworth H., Thomas I. (2018). Assessing graduate sustainability capability post-degree completion: Why is it important and what are the challenges?. Int. J. Sustain. High. Educ..

[52] UNESCO (2017). Education for Sustainable Development Goals: Learning Objectives.

[53] Bielefeldt A.R. (2013). Pedagogies to Achieve Sustainability Learning Outcomes in Civil and Environmental Engineering Students. Sustainability.

[54] Bleys B., Defloor B., Van Ootegem L., Verhofstadt E. (2018). The Environmental Impact of Individual Behavior: Self-Assessment Versus the Ecological Footprint. Environ. Behav..

[55] Chen S.T., Chang H.T. (2016). Factors that affect the ecological footprint depending on the different income levels. AIMS Energy.

[56] Bayraktar S. (2020). Factors Contributing Ecological Footprint Awareness of Turkish Pre-Service Teachers. Int. Educ. Stud..

[57] Gündüz S., Alsagher E.A.A. (2018). Consciousness levels of Libyan higher education students on ecological footprint and sustainable life. Qual. Quant..

